# Supplementary material for: Morphodynamical cell state description via live-cell imaging trajectory embedding
Source: Commun Biol. 2023 May 4;6:484. doi: 10.1038/s42003-023-04837-8 (PMC10160022; doi:10.1038/s42003-023-04837-8)
Supplement: Supplementary file 1 — Supplementary Information [file 42003_2023_4837_MOESM1_ESM.pdf]

Morphodynamical cell state description via live-cell imaging trajectory embedding

Authors: Jeremy Copperman[1,*], Sean M. Gross[1], Young Hwan Chang[1,2], Laura M. Heiser[1,2,*] and Daniel M. Zuckerman[1,*]

Affiliations:

[1] Department of Biomedical Engineering, Oregon Health and Science University, Portland OR 97239, U.S.A.

[2] Knight Cancer Institute, Oregon Health and Science University, Portland OR 97239, U.S.A

* corresponding authors (copperma@ohsu.edu, heiserl@ohsu.edu, zuckermd@ohsu.edu)

Supplementary Figures and Tables

Supplementary Table 1. Segmentation and tracking manual validation. 100 cells per treatment were randomly selected, and evaluated by eye to qualitatively assess segmentation and tracking accuracy. Fraction segmented was estimated by the image area covered by segmented masks divided by the area selected as being occupied by cells from the ilastik random forest pixel classifier. *(+EGF)

Ligand	total	EGF	HGF	OSM	IFNG*	BMP2*	TGFB*
% segmented	52%	53%	51%	46%	54%	52%	42%
% good seg	43%	52%	38%	33%	48%	53%	56%
% bad seg	26%	26%	24%	7%	25%	33%	34%
%ambiguous seg	31%	22%	38%	60%	27%	14%	10%
% tracked	60%	51%	56%	55%	60%	58%	79%
% good tracks	87%	95%	91%	42%	94%	96%	95%
% bad tracks	2%	5%	0%	3%	6%	0%	0%
%ambiguous tracks	11%	0%	9%	55%	0%	4%	5%

Supplementary Figure 1. Segmentation and tracking manual validation. Examples of good, bad, and ambiguous qualitative validation categories for segmentation and tracking.

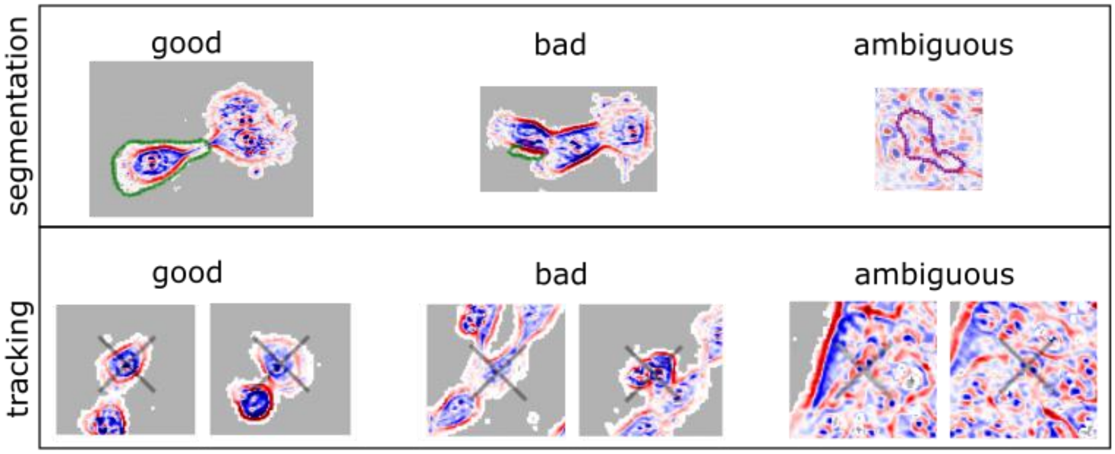

Supplementary Table 2. Number of extracted trajectory snippets with increasing snippet length. *(+EGF)

Trajectory snippet length	PBS	EGF	HGF	OSM	BMP2*	IFNG*	TGFB*
1	49016	73093	69747	99933	63669	72389	49008
2	32251	35337	40640	58119	31558	39545	36736
4	21894	14066	22837	29783	14135	19501	16206
8	15342	4892	12572	13208	5772	9033	2936
16	9256	1416	5857	3861	1892	3603	884
32	4243	226	2161	924	409	1055	142
64	1002	16	347	139	0	141	0

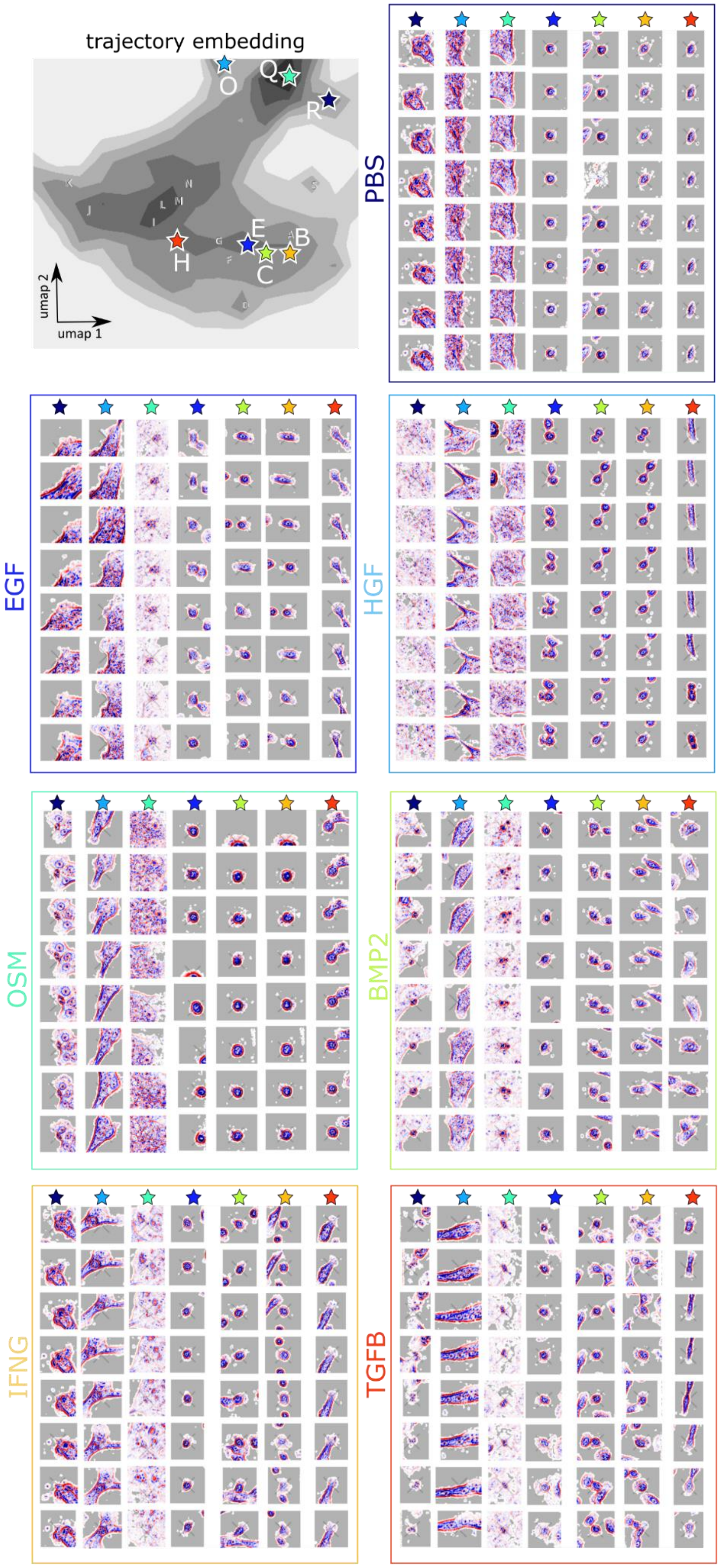

Supplementary Figure 2: Trajectory embedding constructs a common space to evaluate unique and shared cell morphodynamics. Top left: outline of the combined density distribution in the trajectory embedding (snippet length = 8) space (gray), with locations of the density peaks in individual treatments marked with letters consistent with Figure 5 and exhibited cell trajectory snippets at locations marked with stars. Remaining boxes: cell trajectory snippet extracted at the marked location, but from the treatment labeled for each box. Time for each 8-step trajectory snippet shown runs from top to bottom.

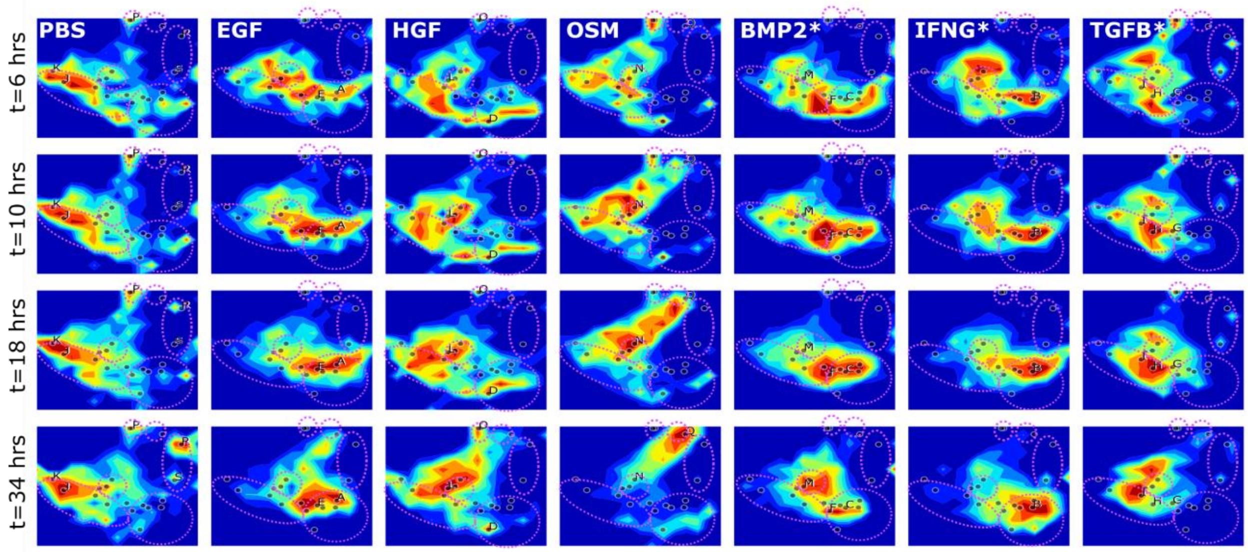

Supplementary Figure 3:
Time-dependent cumulative distributions (rainbow) from 12-hr windowed averages (or maximum allowed window average), and density peak locations selected as fine-grained metastable states (labels A-S).
*(+EGF)

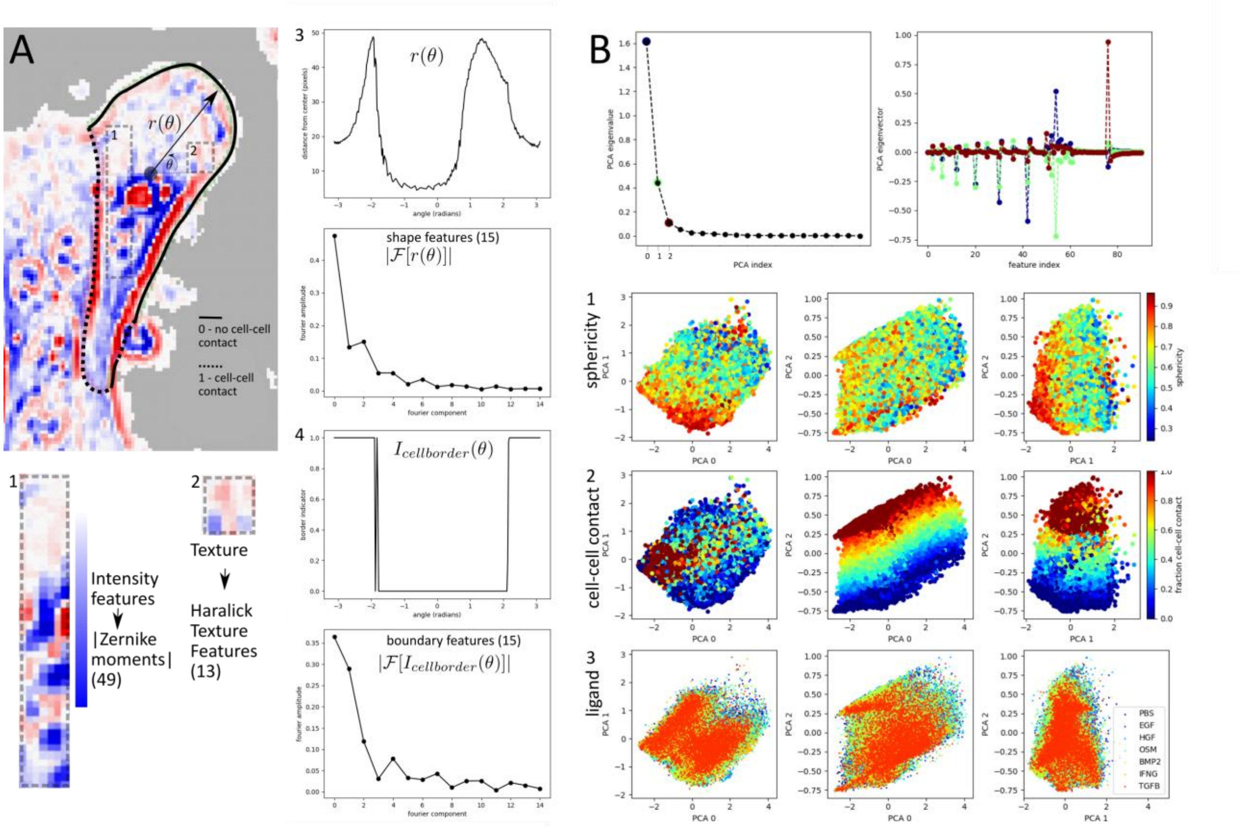

Supplementary Figure 4:
Cell features and PCA reduction. A) Segmented single-cell with cell border in contact with another cell (dashed line), and not in contact (solid line), and cell feature description 1. Phase contrast overall features described by Zernike moment absolute values. 2. Texture described by Haralick texture features. 3. Cell shape featurized by the absolute value of the Fourier coefficients of the distance to the cell center as a function of the angle θ. 4. Local cell environment described by the absolute value of the Fourier transform of $I(\theta)$ indicating cell-cell contact. B) PCA feature reduction. PCA eigenvalues (top left) and eigenvectors for the top 3 components explaining >90% of the feature variance (top right). PCA landscape colored by 1. Approximate sphericity given by the first Fourier component of $r(\theta)$, approximate fraction of the cell boundary in contact with another cell given by the first Fourier component of the cell-cell border indicator $I(\theta)$, and 3. by ligand condition.

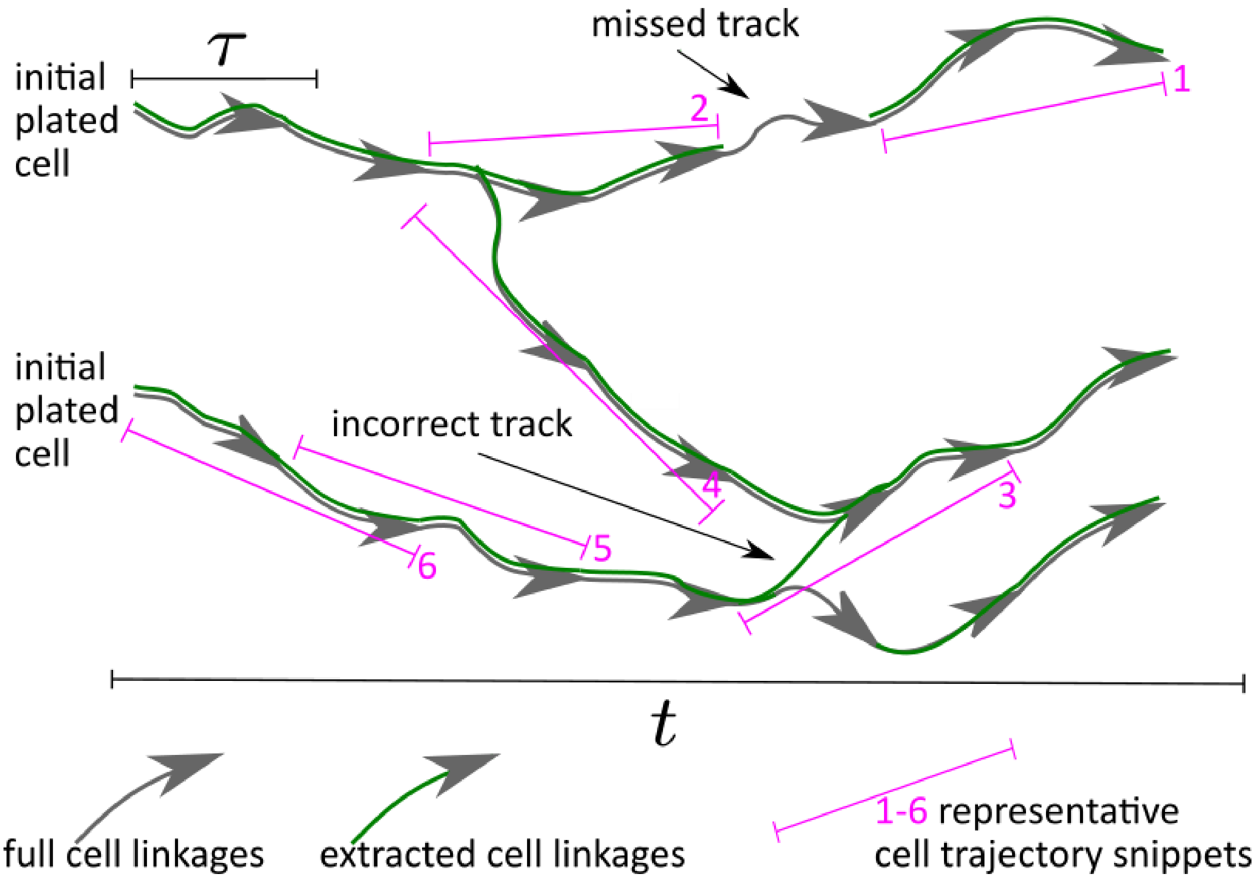

Supplementary Figure 5:
Graphical illustration of the full set of cell linkages connecting 2 initially plated cells to the cells at the final timepoint (gray arrows). The extracted cells and linkages from the cell segmentation and tracking steps as green arrows indicating the available partial set of cells and linkages used in the data analysis with errors and missing cells, and some possible trajectory snippets (yellow highlights) extracted in a sliding window manner along the extracted linkages.

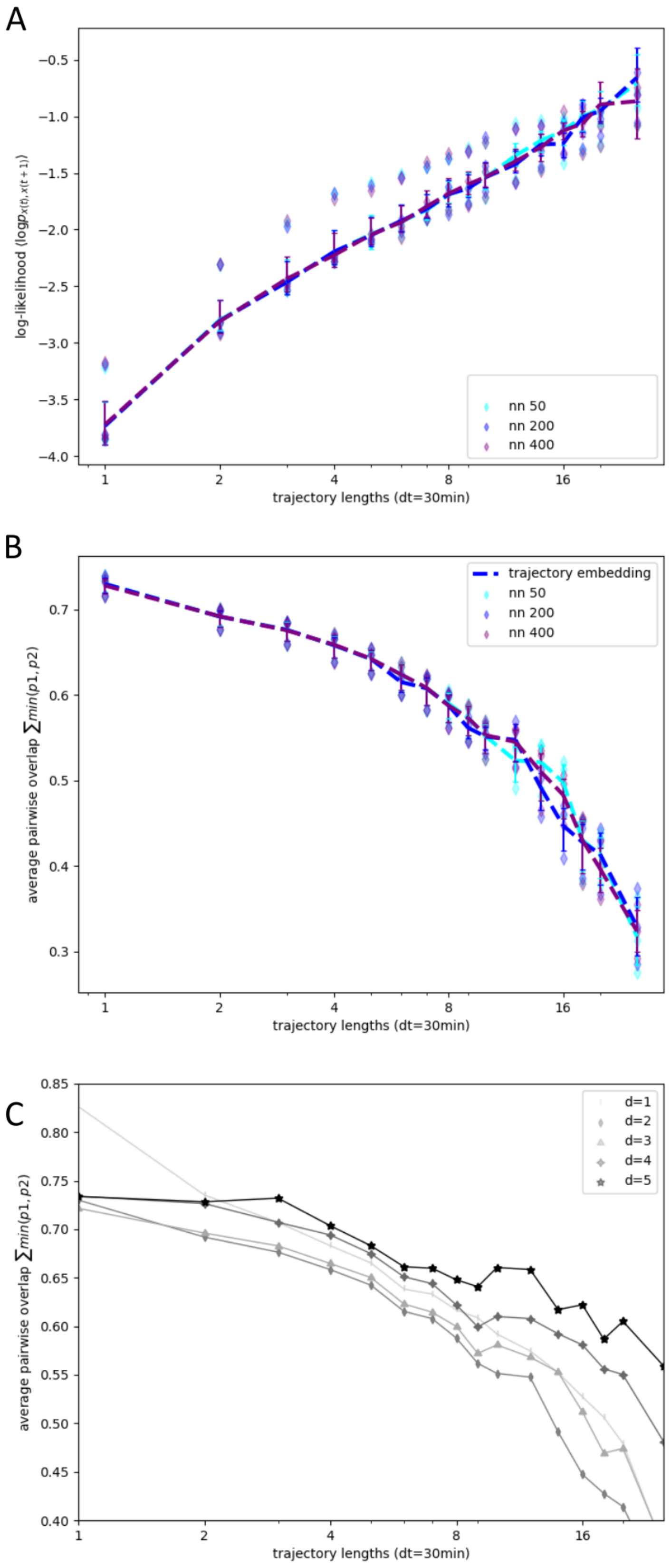

Supplementary Figure 6:
A) Trajectory log-likelihood with trajectory length at half the value of the UMAP n_neighbors parameter (nn=50, cyan) and twice the value of the n_neighbors parameter (nn=400, purple), and the value used in Figures 2-5 (nn=200). B) Average pairwise overlap between ligand population distributions at half the value of the UMAP n_neighbors parameter (nn=50, cyan) and twice the value of the n_neighbors parameter (nn=400, purple), and the value used in Figures 2-5 (nn=200). C) Average pairwise overlap between ligand population distributions with UMAP embedding dimension (d=1-5, light-gray to black).
