# Supplementary material for: Morphodynamical cell state description via live-cell imaging trajectory embedding
Source: Commun Biol. 2023 May 4;6:484. doi: 10.1038/s42003-023-04837-8 (PMC10160022; doi:10.1038/s42003-023-04837-8)
Supplement: Supplementary file 3 — Editorial Assessment Report [file 42003_2023_4837_MOESM3_ESM.pdf]

nature portfolio GUIDED OPEN ACCESS

Contents of this report

1. **Manuscript details**: overview of your manuscript and the editorial team.
2. **Review synthesis**: summary of the reviewer reports provided by the editors.
3. **Editorial recommendation**: personalized evaluation and recommendation from all 3 journals.
4. **Annotated reviewer comments**: the referee reports with comments from the editors.
5. **Open research evaluation**: advice for adhering to best reproducibility practices.

About the editorial process

Because you selected the **Nature Portfolio Guided Open Access** option, your manuscript was assessed for suitability in three of our titles publishing high-quality work in your field of research: ***Nature Computational Science, Nature Communications, and Communications Biology.*** More information about Guided Open Access can be found here.

Collaborative editorial assessment

Your editorial team discussed the manuscript to determine its suitability for the Nature Portfolio Guided OA pilot. Our assessment of your manuscript takes into account several factors, including whether the work meets the **technical standard** of the Nature Portfolio and whether the findings are of **immediate significance** to the readership of at least one of the participating journals in the Guided OA pilot.

Peer review

Experts were asked to evaluate the following aspects of your manuscript:

- **Novelty** in comparison to prior publications;
- **Likely audience** of researchers in terms of broad fields of study and size;
- **Potential impact** of the study on the immediate or wider research field;
- **Evidence** for the claims and whether additional experiments or analyses could feasibly strengthen the evidence;
- **Methodological detail** and whether the manuscript is reproducible as written;
- Appropriateness of the **literature review**.

Editorial evaluation of reviews

Your editorial team discussed the potential suitability of your manuscript for each of the participating journals. They then discussed the revisions necessary in order for the work to be published, keeping each journal's specific editorial criteria in mind.

Journals in the Nature portfolio will support authors wishing to transfer their reviews and (where reviewers agree) the reviewers' identities to journals outside of Springer Nature.
If you have any questions about review portability, please contact our editorial office at guidedoa@nature.com.

nature portfolio

GUIDED OPEN ACCESS

Manuscript details

Tracking number	Submission date	Decision date	Peer review type
GUIDEDOA-22-00464	Apr 20, 2022	Jun 23, 2022	Single-blind

Manuscript title

Morphodynamical cell-state description via live-cell imaging trajectory embedding

Preprint: bioRxiv

Author details

Dr. Jeremy Copperman

Affiliation: Oregon Health & Science University

Editorial assessment team

**Primary editor**	**Gene Chong**<br>**Home journal:** *Communications Biology*<br>**ORCID:** 0000-0001-7250-4046<br>**Email:** gene.chong@us.nature.com
**Other editors consulted**	**Ananya Rastogi**<br>**Home journal:** *Nature Computational Science*<br>**ORCID:** 0000-0003-3030-8535<br><br>**Aline Lückgen**<br>**Home journal:** *Nature Communications*<br>**ORCID:** 0000-0001-6136-7592
**About your primary editor**	Gene received his PhD in Chemistry from Johns Hopkins University and did his postdoctoral research at the University of Maryland, Baltimore. He has research experience in molecular dynamics simulation methods and applications in nanotechnology, glycobiology, and antibody design. Gene joined the editorial team of *Communications Biology* in August 2021 and handles papers in computational biology, biochemistry, biophysics, and structural biology.

Editorial assessment and review synthesis

**Editor's summary and assessment**	Most analyses of live-cell image data have been primarily based on a static classification of cell morphology and do not directly classify the dynamic landscape of cell morphology trajectories. The authors develop a generalizable morphodynamical trajectory embedding method that can be used to analyze live-cell imaging datasets composed of unlabeled phase-contrast microscopy images as well as multiplexed imaging with molecular reporters.<br><br>The primary editor decided to send this manuscript out to review for the development of an analysis method that incorporates morphodynamical trajectories and the demonstration that it improves the prediction of cell states over existing methods analyzing static morphological snapshots.
**Editorial synthesis of reviewer reports**	All reviewers find the work novel and potentially influencing thinking in the specialist field. They mainly ask for more clarification or elaboration with no additional experiments.<br><br>The editors all agree that the reviewers' comments are positive, and *Communications Biology* would be happy to invite a suitably revised manuscript addressing all reviewer comments to consider for publication.<br><br>The editors at *Nature Computational Science* and *Nature Communications* believe that the novelty in computational methods and striking biological insight are limited to appeal to a broader audience in their respective journals and do not invite revisions.

Editorial recommendation

Nature Computational Science<br><br>Revision not invited	While the application of the multiple methods is interesting and solves a challenging problem in the field, this is out of scope for NCS since the authors use previously established tools, and therefore revision is not invited for this journal.
Nature Communications<br><br>Revision not invited	Revision is not invited at this journal due to several limitations raised during the editorial assessment, including the limited computational novelty, the focus on a single cultured cell type and the lack of striking biological insights.
Communications Biology<br><br>Major revisions	Please address all comments raised by reviewers and ensure that all additional methods details, discussion, and analysis are performed as requested.

Next steps

**Editorial recommendation 1:**	Our top recommendation is to revise and resubmit your manuscript to *Communications Biology.* We feel the additional experiments required are reasonable to address within a 6-month timeframe.
**Note**	As stated on the previous page *Nature Computational Science* and *Nature Communications* are not inviting a revision at this time. Please keep in mind that the journal will not be able to consider any appeals of their decision through Guided Open Access.

Revision

To follow our recommendation, please upload the revised manuscript files using **the link provided in the decision letter**. Should you need assistance with our manuscript tracking system, please contact Adam Lipkin, our Nature Portfolio Guided OA support specialist, at guidedOA@nature.com.

Revision checklist

- ☐ Cover letter, stating to which journal you are submitting
- ☐ Revised manuscript
- ☐ Point-by-point response to reviews
- ☐ Updated Reporting Summary and Editorial Policy Checklist
- ☐ Supplementary materials (if applicable)

Submission elsewhere

If you choose not to follow our recommendations, you can still take the reviewer reports with you.

Option 1: Transfer to another Nature Portfolio journal
Springer Nature provides authors with the ability to transfer a manuscript within the Nature Portfolio, without the author having to upload the manuscript data again. To use this service, **please follow the transfer link provided in the decision letter.** If no link was provided, please contact guidedOA@nature.com.

> *Note that any decision to opt in to In Review at the original journal is not sent to the receiving journal on transfer. You can opt in to In Review at receiving journals that support this service by choosing to modify your manuscript on transfer.*

Option 2: Portable Peer Review option for submission to a journal outside of Nature Portfolio
If you choose to submit your revised manuscript to a journal at another publisher, we can share the reviews with another journal outside of the Nature Portfolio if requested. You will need to request that the receiving journal office contacts us at guidedOA@nature.com. We have included editorial guidance below in the reviewer reports and open research evaluation to aid in revising the manuscript for publication elsewhere.

Annotated reviewer reports

The primary editor has included some additional comments on specific points raised by the reviewers below, to clarify requirements for publication in the recommended journal. However, please note that all points should be addressed in a revision, even if an editor has not specifically commented on them.

Reviewer #1 information	
**Expertise**	This reviewer has expertise in computational biology and live cell imaging analysis.
**Editor's comments**	This reviewer finds the work to be novel. The reviewer has similar concerns with other reviewers that methods are lacking in detail and that the Github repo is missing clear README documentation and sample datasets for reproducibility.

Reviewer #1 comments	
**Section**	**Annotated Reviewer Comments**
**Remarks to the Author: Overall significance**	The manuscript provides framework that utilize cell motion to embed shape features in the shared space. It will make the morphodynamical state of the cell easier to calculate and compare. The result is quite novel and can help to extend the work in the cell shape and motility research. Other research that quite similar is "Integrated Analysis of the cell shape and movement in moving frame" by Heryanto et al. However, the Heryanto et. al. focused in 3D shape.
**Remarks to the Author: Impact**	In my opinion, this manuscript has impact in the live cell, cell shape, and cell movement research. There are growing data and studies in this area. However, we still lack the computational methods to investigate and analyze these data.
**Remarks to the Author: Strength of the claims**	The methodology of the work is reasonable and has biological plausibility. I have some question regarding the methodology:<br><br>1. What is the reason why zernike moment, haralick texture features, and absolute value of fourier transform were selected? What happen if I choose the different shape features such as mentioned in Robert Osada paper (2002) "Shape Distributions" or wavelet as used in "Wavelet descriptor of planar curves: Theory and applications"?. The introduction, discussion, and reference about these shape/morphodynamical features should be included because the manuscript title itself contain "Morphodynamical cell-state" terms.<br><br>2. What does it mean cell lineages here? Is it the developmental/differentiation history of the cell? Or the cell movement tracking history? No clear definition here<br><br>3. The UMAP parameter should be mentioned in the methods section. UMAP is a

	stochastic algorithm so UMAP will produce different results depending on the initial value or parameters. How the authors decide which UMAP result will be used in the manuscript? Is there any effect of the different parameters of UMAP to the result of the experiment?<br>**Testing parameters and justifying the choice of parameter will be necessary for further consideration at *Communications Biology.***<br><br>4. The authors wrote "We found minimal ligand-specificity in the embedding space of morphological snapshots, with increased ligand specificity observed in the embedding space of morphological trajectories". How does the author measure the ligand specificity here? If possible please write the number/measurement to increase the clarity.<br><br>5. The author wrote, "$T = Cij/Ci$ and $Ci = \sum j\ Cij$" . Please define the Cij and Ci here. Also in trajectory likelihood, please define Tij . Is the Tij it same T as in T= Cij/ Ci?
**Remarks to the Author: Reproducibility**	To be honest the methodology section is confusing especially in the subsection: 'Cell featurization', 'cell state clustering and prediction', and 'cell metastable state extraction and grouping'. If the author can provide the diagram, algorithm step, or supplemental page, it will improve the clarity and reproducibility.<br><br>The github source code of this paper should have any Readme and instructions how to replicate the experiment. It should also contain some data (real or toy data) to replicate the experiment<br>**Please include a README and datasets in the Github repo to replicate the experiments. This is essential for open access and code availability policies in the Nature Portfolio.**

Reviewer #2 information

**Expertise**	This reviewer has expertise in computational biology and live cell imaging analysis.
**Editor's comments**	This reviewer finds the work novel but also has concerns with method details and code and data accessibility.

Reviewer #2 comments

**Section**	**Annotated Reviewer Comments**
**Remarks to the Author: Overall significance**	Copperman et al. provide a novel analytical approach to studying live-cell imaging data. Rather than analyzing each snapshot in time independently, they instead create a "morphodynamical trajectory", which includes the time component in single cell featurization. Using an example perturbation dataset, they demonstrate that incorporating time better distinguishes the phenotypic consequences of the perturbations and more readily identifies cell state trajectories. Overall, the authors accurately attribute previous work, the method is quite novel and seems to work well, and the results are beautifully visualized. However, there are several elements in the methods and results that should be clarified prior to acceptance.
**Remarks to the Author: Impact**	Yes, I believe the paper will influence thinking about live-cell imaging featurization.
**Remarks to the Author: Strength of the claims**	Yes, the work is convincing. I do not believe any additional experiments are required, but I do believe the authors need to clarify many major and minor elements. I outline these concerns as follows:<br><br>Major elements:<br>1. The authors need to discuss how they handled cell division events. How often do the cells divide over the time course? Do multiple daughter cells share the same embedding prior to the time point they divided? Do the authors perform any synchronization across cell cycle stage? Is directionality toward the attractor state 100% consistent for all cells, and for all cells no matter the cell cycle stage?<br><br>2. In the methods section, the authors state: "Experimental protocols can be found in detail at the publicly available Synapse database38." The citation number 38 refers to a preprint that cites the synapse database. Given the paper describes the data thoroughly, it is a sufficient resource. However, the synapse database requires a sign-in to view the methods. There should be no sign-in restrictions to view the experimental design, and the present paper should at

least describe the critical methodological details, in the event the resource is deprecated and lost. For example, Was ligand treatment concurrent with imaging? When was the ligand introduced?

Please make datasets available without sign-in restrictions.

3. The authors should better introduce the concept of trajectory length, and what they hope to achieve by testing multiple lengths – did their analyses reveal an optimal length? Why not use the full length? Some of this was described in the results section of Figure 4B, but this should be revised and elaborated on. For example, the authors state: "We expect in general that greater trajectory embedding lengths will increase the descriptive capability of trajectory models, but only up to the point where adequate data quantity is obtained." How can a reader determine when adequate data is obtained?

Minor elements

4. I recommend that the authors provide a more detailed description of the concept of "ligand perturbation". The methods should provide much more detail on how the ligands were prepared and applied.

5. On Page 4, the authors state: "Comparing morphodynamical trajectories between the different ligand treatments requires the construction of a shared cell-state space which we created by embedding the morphodynamical tensors of cells under all of the ligand treatment conditions together." Embedding is a confusing word to use in this context, especially when discussing "embedding space" in the next sentence. Do the authors mean concatenating?

6. More detail is required in extended figure 2 – what do the colors of the images represent? What is the scale? Each image going down the rows represents 30 minute intervals? (the colors are labeled in figure 1, but I still don't know what red+ and blue- mean. This is described briefly in the methods, sufficiently. But this should be introduced at least a little to reduce confusion elsewhere.)

7. Is Figure 2B depicting all snapshots, or just the first time point? All cell trajectory length of 8, what about cells with longer trajectory lengths?

8. Figure 2C – The error bars represent a 95% bootstrapped confidence interval of three data splits – what are the data splits? The x axis scale is strange – the authors should point this out. The null embedding model should be described in a bit more detail in the legend.

9. On Page 5, the authors state: "Ligand perturbation induces time-dependent morphologic and phenotypic differences in the cellular populations." – what do the authors mean by phenotypic differences other than morphology?

10. "To compare cell states and pathways, we took trajectories that were longer than the morphodynamical tensor embedding length (3.5 hrs) and projected

	them onto the snippet-based landscape.” – The authors should clarify what they mean by “took”. Also, it is unclear how there could be trajectories longer than the embeddings? How did the authors project them?<br><br>11. The authors should clarify how they selected the example single cells in Figure 3.<br><br>12. The readers would benefit from a note on computational efficiency – this analysis is performed using 3 PCs only – can this scale to more PCs? What do these PCs represent? Do all ligands separate based on 3 PCs?<br><br>13. The authors introduce nonstandard math in page 8 – what do the <> brackets represent (e.g. sqrt(<(x-<x>)^2>)?<br><br>14. In figure 4, what does variable d represent? I’m having a difficult time interpreting why a decreasing locality ratio can be interpreted as increasingly systematic. Does this mean that treatment with ligand makes trajectory representations converge to similar attractor states? Figure 4 is also the first time I’m noticing that there is combination treatments? This should be introduced sooner.<br><br>15. What evidence of a mesenchymal-like morphological state do the authors use for intermediates? A reader requires more annotation details in Figure 5 (epithelial-like, intermediate, bound, budding, etc.) How did the authors derive these? Ah, I see, in the methods, “cell state names are descriptive for ease of interpretation, but not based upon validated biological interpretation” – in this case, then I think the names offer more confusion than ease of interpretation. If the annotations are kept, then a description of how they were derived should coincide with their introduction.<br><br>16. “Cells were mapped into these macrostates by first finding the closest metastable state (Figure 5 A.-S.)“. The authors likely mean Figure 5B A.-S.<br><br>17. “Principal components of each cell were aligned, and then single-cell features were calculated.” – What does it mean for the principal components to align?<br><br>18. Cells were tracked by “minimum distance” – what does minimum distance mean?
**Remarks to the Author: Reproducibility**	The Github repository is nice to see, however, the README should document each analysis step and clearly describe how to reproduce the analysis. I cannot determine reproducibility from the Github repo as presented, as it requires substantially more documentation.

	The study links a biorxiv preprint for data access, but specific access to the data should also be provided in the paper, and download instructions should be provided in the github.<br><br>Confirming a reproducible github repository, clarifying data access, and increasing detail of many methodological elements, as outlined above, must be performed in order for the paper to maximize reproducibility.<br>**Again, this a requirement and echoes Reviewer #1's comments.**

Reviewer #3 information

**Expertise**	This reviewer has expertise in computational biology and cellular morphodynamics.
**Editor's comments**	This reviewer finds the work novel but the application is less convincing or at least needs improved presentation and explanation. The text needs restructuring. As the Methods section comes at the end of the text, key details forming the methods need to be introduced earlier for a linear read.

Reviewer #3 comments

Section	Annotated Reviewer Comments
**Remarks to the Author: Overall significance**	In this manuscript, the authors present a methodology for characterizing morphological states of cells and their change over the course of live-cell imaging. The method is based on extracting cell outlines and trajectories, characterizing each cell with 92 metrics (including textural, shape, and near-neighborhood features), and mapping these metrics on a cell-state landscape using the dimension reduction technique UMAP (Uniform Manifold Approximation and Projection). The novelty of the presented approach is in extending the input vector for UMAP algorithm by including cell features from multiple time points (specifically, from the running time window of a chosen length). The idea of such "trajectory embedding methodology" was applied previously, but not in the context of cellular morphodynamics.<br><br>The authors tested their novel methodology on a previously reported imaging dataset from the MCF10A (normal human mammary epithelial cells) project within the LINCS (Library of Integrated Network-Based Cellular Signatures) consortium.<br><br>The authors analyzed cell-state landscapes of MCF10A cells subjected to six ligand treatments and demonstrated that trajectory-based UMAP increases the distinguishability of cell-states and predictability of cell trajectories in the morphological landscapes for different treatments as opposed to the approach based on feeding UMAP with individual time-point features sets.<br><br>Finally, the authors interpreted their cell-state transition network analysis with a conclusion that cell-cluster formation stimulated by the treatments follows through an intermediate mesenchymal-like state (although I find this part of the results a bit less convincing).<br>**Please make sure that conclusions are fully supported by data and address Reviewer #3's comments below on "Strength of the claims".**

	Overall, this work is an important development in the field of high-throughput cell characterization, can be generalized to include molecularly labeled imaging, transcriptomic, and proteomic data, and should be of a high interest to researchers working on drug discovery or other perturbation screenings. The manuscript does have a sufficiently detailed introduction to the topic with appropriate citations and discussions of prior studies.
**Remarks to the Author: Impact**	The methodology presented in this manuscript was tested on phase-contrast microscopy images with a pipeline that relies on cell segmentation, which is a long-standing challenge for the fully automated methods of cell shape extraction from that kind of images. The authors argued that their "trajectory embedding analysis allows for the robust and systematic characterization of cell state trajectories even in this challenging data analysis regime with many missing and partially segmented cells". If true, such methodology should have a significant impact in the field and stimulate further discussions, tests, and refinements.<br><br>Even beyond image analysis issues, a reliable method of identification of cell-states and their dynamic relationships during a biological process is of fundamental interest in the biomedical research.
**Remarks to the Author: Strength of the claims**	Overall, the authors' claims are well-supported and illustrated with quantitative metrics. However, the clarity of the presentation could be significantly improved. Below, I list my concerns and suggestions to consider for a revised version of the manuscript:<br><br>1. The key concept of this manuscript is the "morphodynamical trajectory embedding", which is explained in sufficient detail only at the end of the manuscript in the Methods section after Discussion and Conclusions. Thus, a reader may struggle with understanding the essence of the approach until the end of the paper or need to skip to the Methods before reading the Results. Even, the phrase "using morphological feature trajectory histories, rather than the common practice of examining morphological feature time courses" in the Abstract may be confusing because 'trajectory histories' and 'feature time courses' sound like the same thing. Because this is mostly a method paper, I think it will be helpful to explain the main concept in the beginning of the Results section. The other methodological details can be left in the Methods section.<br><br>2. End of Page 2: "Image sequences of embedded cell trajectory windows (snippets) mapped to the same location of the trajectory embedding space share morphodynamical features across treatments, see extended figure 2." Is this a qualitative statement based on the visual inspection of the selected cells shown in Extended Figure 2? Or is this just a natural consequence of the way the trajectory embedding space is constructed? In other words, I am not sure

how to assess the agreement between morphological changes within snippets under different treatments using Extended Figure 2.

3. Figure 1C. Zernike moments (49 features) are referred here as ‘global’. Global in what sense? Should these features be called Zernike moments just to be clear and specific?

4. Figure 1E. Green, red, and blue segments are not defined. It is not clear what they are supposed to represent.

5. Figure 1F. This is the first time ‘embedding’ is defined as UMAP. I think this should be stated early in the text. Furthermore, it would be helpful to provide some very basic introduction to the UMAP technique (in addition to a citation).

6. Page 6. “The pairwise overlap decreased more rapidly than in a null model where the cell features were randomly scrambled within treatment (Figure 2C).” In Methods, the overlap for a pair of distributions is briefly defined as a sum of minimal values (also indicated as the vertical axis label in the figure). However, there are 28 pairs of treatments. Does Figure 2C show the average over 28 pairs? If not, which overlap is shown in Figure 2C?

7. Page 8. Second paragraph. The formula for a locality ratio is not clearly defined. Is ‘l’ a function of time? MSD is usually a function of time. If not, there must be an averaging over time in addition to averaging over trajectories. Is ‘x’ in the denominator a function of time? Anyway, please define all functions and averages in the formula.

8. Figure 4A. ‘d’ must be defined (preferably in the part that introduces UMAP).

9. Section “Morphodynamical transitions precede cell-cluster formation”. It is not clear to me based on what local peaks in the cell-state landscape are combined into 6 macrostates. For example, which clusters are bound, and which are unbound? ‘Bound’ in what sense? What is termed ‘budding’? Figure 5B doesn’t help to understand the authors’ decision process. I have hard time visually distinguishing ‘E’ and ‘F’ in the epithelial-like macrostate from ‘G’ and ‘H’ in the mesenchymal-like macrostate.

Furthermore, the description in the text creates an expectation that during the 48-hour observation treated cells tend to transition from separated cells (macrostate 1) to tight clusters (macrostates 4 and 5) through the mesenchymal-like macrostate 2. However, the networks in Figure 5A show that dominant directions of the flow are mostly from 4 to 3 and from 3 to 2. I understand that the only way to transition from 1 to 4 and 5 is through the intermediate states 2 and 3, but why are the flows stronger in the opposite direction?

	I don't see any 'white arrows' in Figure 5C as mentioned in the figure caption.<br><br>I think this section requires some reworking and additional explanations.<br><br>10. Methods. Section "Cell segmentation". What does it mean "segmentation was performed iteratively over multiple rounds"? Multiple rounds with progressively larger estimated cell size?<br><br>"(pixel values of segmented cells set to 0)". Why 0? Should it be 1?<br><br>11. Methods. Section "Cell featurization". "Shape features (15 features) were calculated as the absolute value of the fourier transform of the distance to the boundary as a function of the radial angle around cell center" I would say coefficients for the first 15 frequencies to be more specific.<br><br>"…with the set of shape features normalized to 1". Do you mean "sum of shape features"?<br><br>12. Methods. Section "Morphodynamical tensor embedding". It is not clear how cell division is treated? Does this initiate two new trajectories? Or trajectories split, so that parts of cell trajectories can overlap? What exactly "partial cell lineages" mean?<br><br>"…with cell histories up to a given length". Do you mean "equal or longer" rather than "up to"?<br><br>"…for each treatment shown extended data table 2". "in" is missing after "shown".<br><br>13. Methods. Section "Stochastic dynamics". Please see comment 7 regarding the locality ratio formula.<br><br>14. Methods. Section "Cell state clustering and prediction". "T = Cij/Ci". Should it be "Tij = Cij/Ci"?
**Remarks to the Author: Reproducibility**	I do not see any issues with the statistical analysis. To ensure reproducibility the authors need to clarify technical concerns in the list above.

Open research evaluation

Data availability
**Data Availability Statement**
Thank you for including a Data Availability statement. Please make it separate from the Code Availability statement. While you have included some important information, the editors and reviewers have noted that some details appear to be missing. The Data Availability Statement should be as detailed as possible and include accession codes or other unique IDs for deposited data, information about where source data can be found, and specify any restrictions to data access that may apply. At a minimum, the statement should indicate that data are available upon request and explain how data access can be granted. If data access is not possible, the reasons for this must be made clear in the Data Availability Statement.<br><br>More information about the Nature Portfolio data availability policy can be found here: https://www.nature.com/nature-portfolio/editorial-policies/reporting-standards#availability-of-data<br><br>Please add the following information to your Data Availability Statement:<br>Sample datasets that can be run with code to replicate experiments as mentioned by reviewers. The synapse database requires sign-in access as mentioned by reviewers. Please comment or resolve alternative open access solutions to datasets. Specify “in some formats” and “additional data”.<br><br>More information about formatting Data Availability Statements can be found here: http://www.springernature.com/gp/authors/research-data-policy/data-availability-statements/12330880
**Other data requests**
In line with community standards regarding open research, Springer Nature strongly supports data sharing and believes that all datasets on which the conclusions of the paper rely should be available to readers. We encourage authors to ensure that their datasets are either deposited in publicly available repositories (where available and appropriate) or presented in the main manuscript or additional supporting files whenever possible.<br><br>To learn more about data sharing and recommended data repositories, please see https://www.springernature.com/gp/authors/research-data-policy/repositories/12327124

Code availability and citation
Thank you for making your custom code available via Github. Upon publication, Nature Portfolio journals consider it best practice to release custom computer code in a way that allows readers to repeat the published results. Code should be deposited in a DOI-minting repository such as Zenodo, Gigantum or Code Ocean and cited in the reference list following the guidelines described in our policy pages (see link below). Authors are encouraged to manage subsequent code versions and to use a license approved by the open source initiative.<br><br>See here for more information about our code availability policies: https://www.nature.com/nature-portfolio/editorial-policies/reporting-standards#availability-of-computer-code
**Reporting & reproducibility**
We believe that research publications should adhere to high standards of transparency and robustness in their methods and results. This, in turn, supports the principle of reproducibility, which is a foundation of good research, especially in the natural sciences.<br><br>The Methods section should contain sufficient detail such that the work could be repeated. It is preferable that all key methods be included in the main manuscript, rather than in the Supplementary Information. Please avoid use of “as described previously” or similar such as “can be found in detail at”, and instead detail the specific methods used, with appropriate attribution.<br><br>Please note that Nature Portfolio journals allow unlimited space for Methods.
